# A comparative study of three conservative treatments in patients with lumbar spinal stenosis: lumbar spinal stenosis with acupuncture and physical therapy study (LAP study)

**DOI:** 10.1186/s12906-018-2087-y

**Published:** 2018-01-19

**Authors:** Hiroyuki Oka, Ko Matsudaira, Yuichi Takano, Daichi Kasuya, Masaki Niiya, Juichi Tonosu, Masayoshi Fukushima, Yasushi Oshima, Tomoko Fujii, Sakae Tanaka, Hirohiko Inanami

**Affiliations:** 1 Department of Medical Research and Management for Musculoskeletal Pain, 22nd Century Medical & Research Center, Faculty of Medicine, University of Tokyo, Hongo 7-3-1, Bunkyo, Tokyo, 113-8655 Japan; 2 Department of Orthopedic Surgery, Inanami Spine and Joint Hospital, Tokyo, Japan; 3 Department of Orthopedic Surgery, Iwai Orthopaedic Medical Hospital, Tokyo, Japan; 4 Acupuncture and Moxibustion Section Central Rehabilitation Service, University of Tokyo Hospital, Tokyo, Japan; 5 Department of Orthopaedic surgery, Kanto Rosai Hospital, Kanagawa, Japan; 6 Department of Orthopaedic Surgery, University of Tokyo, Tokyo, Japan

**Keywords:** Lumbar spinal stenosis, Conservative management, Medication, Exercise, Acupuncture, Zurich claudication questionnaire

## Abstract

**Background:**

Although the efficiency of conservative management for lumbar spinal stenosis (LSS) has been examined, different conservative management approaches have not been compared. We have performed the first comparative trial of three types of conservative management (medication with acetaminophen, exercise, and acupuncture) in Japanese patients with LSS.

**Methods:**

Patients with L5 root radiculopathy associated with LSS who visited our hospital for surgical treatment were enrolled between December 2011 and January 2014. In this open-label study, patients were assigned to three treatment groups (medication, exercise, acupuncture) according to the visit time. The primary outcomes were Zurich claudication questionnaire (ZCQ) scores before and after 4 weeks of treatment. Least square mean analysis was used to assess the following dependent variables in the treatment groups: changes in symptom severity and physical function scores of the ZCQ and the ZCQ score of patient’s satisfaction after treatment.

**Results:**

Thirty-eight, 40, and 41 patients were allocated to the medication, exercise, and acupuncture groups, respectively. No patient underwent surgical treatment during the study period. The symptom severity scores of the ZCQ improved significantly after treatment in the medication (*p* = 0.048), exercise (*p* = 0.003), and acupuncture (*p* = 0.04) groups. The physical function score improved significantly in the acupuncture group (*p* = 0.045) but not in the medication (*p* = 0.20) and exercise (*p* = 0.29) groups. The mean reduction in the ZCQ score for physical function was significantly greater for acupuncture than for exercise. The mean ZCQ score for treatment satisfaction was significantly greater for acupuncture than for medication.

**Conclusions:**

Acupuncture was significantly more effective than physical exercise according to the physical function score of the ZCQ and than medication according to the satisfaction score. The present study provides new important information that will aid decision making in LSS treatment.

**Trial registration:**

This study was registered with the UMIN Clinical Trials Registry (UMIN000006957).

## Background

Lumbar spinal stenosis (LSS) is usually associated with neurological symptoms and intermittent claudication in the lower extremities due to narrowing of the intervertebral foramen and spinal canal [1]. Because of these symptoms, LSS is an important risk factor for decreased quality of life (QOL), particularly in the elderly. Previous epidemiological studies revealed the prevalence of symptomatic LSS in Japanese individuals ≥ 70 years old of approximately 10% [2]. With aging of the society, the number of patients with LSS is predicted to rapidly increase.

Since rapid neurologic progression and the cauda equina syndrome are rarely observed, the initial treatment for LSS is not surgical intervention but conservative management [3]. The most common type of conservative management is with medications such as nonsteroidal anti-inflammatory analgesics (NSAIDs), with additional administration of gabapentin, pregabalin, and limaprost [4–6]. Pregabalin (PGB; (S)-3-(aminomethyl)-5-methylhexanoic acid) is a new-generation gabapentinoid that binds to the α2δ subunit of the voltage-dependent calcium channel in the central nervous system. PGB was effective in relieving symptoms of LSS in a preliminary prospective study [6].

A randomized controlled trial revealed that not only NSAIDs and a combination of NSAIDs with limaprost, but also limaprost alone were effective in relieving symptoms of LSS in the short term [4, 5]. Although NSAIDs were used in the above study, acetaminophen is usually recommended for safety purposes since patients with LSS are generally in their late years and at a greater risk of the adverse reactions associated with NSAIDs [7, 8].

Exercise is considered to be safe for the elderly and can be cost effective, but its impact in LSS has not been sufficiently studied [9]. Clinical experience suggests that acupuncture in the lumbar region can be an effective treatment for conditions that are associated with LSS [10].

Although a number of studies have examined the efficiency of conservative management of LSS [4–10], different types of conservative approaches have not been compared, and no evidence is available to guide the selection of an appropriate method.

We hypothesized that all three conservative treatments improve the symptoms of LSS, but they are superior or inferior. The present study is the first comparative trial of conservative management approaches of three types (medication [acetaminophen], exercise, and acupuncture) in Japanese patients with LSS.

## Methods

### Study design

Among radiculopathy of LSS, L5 root disorders have been reported to the highest prevalence. [11] In clinical study, it is necessary for quick recruitment and subjects to be homogeneous. Thus the subject was limited to L5 root radiculopathy. Patients with L5 root radiculopathy associated with LSS who visited Iwai Orthopaedic Medical Hospital for surgical treatment between December 2011 and January 2014 were enrolled based on the following eligibility criteria: 1) age: 50–79 years, 2) posture-dependent symptoms of L5 nerve radiculopathy in the gluteals or lower extremities predominantly on one side or typical neurogenic intermittent claudication [12], 3) radiological findings explaining the symptoms, and 4) nerve root type/mixed type LSS. Mixed type LSS has both radicular and caudal type symptoms. In other words, we selected patients with L4/5 lateral recess stenosis or L5/S1 foraminal/lateral stenosis with L5 root radiculopathy. Sagittal lumbar MRI images and lumbar vertebra CT (sagittal, frontal, transverse) images were used for diagnosis. The exclusion criteria were as follows: 1) typical cauda equina symptoms only, 2) significant muscle weakness (tibialis anterior, peroneal muscle, or extensor hallucis longus Manual Muscle Testing (MMT) score < 3), 3) positive straight leg raise test, 4) uncontrolled diabetes mellitus (HbA1C ≥ 7.0), 5) use of panaldin or warfarin (biaspirin could be used), 6) use of depressants or psychotropics, 7) concomitant symptomatic myelopathy (comprehensive judgment based on images of the cervical vertebra and other examination findings), 8)peripheral artery disease (ankle brachial pressure index (ABI) < 0.9), 9) rheumatoid arthritis, and 10) hemodialysis. In this open-label study, patients who meet the inclusion and exclusion criteria were assigned to one of the three groups (in a 1:1:1 ratio): medication, exercise, acupuncture according to the visit time. No patient underwent surgical treatment during the study period. On the statistical analysis section, we described that one group of sample size is about 40 cases. We recruited patients who met inclusion and exclusion criteria in order of medication, exercise, acupuncture until each group reaches approximately 40. The duration of the recruitment was medication, exercise, acupuncture, which were December 2011 to August 2012, September 2012 to March 2013, and April 2013 to December 2013, respectively. The study is being undertaken in compliance with the ICH Good Clinical Practice guidelines (https://www.ich.org/fileadmin/Public_Web_Site/ICH_Products/Guidelines/Efficacy/E6/E6_R1_Guideline.pdf) and the ethics principles set out in the Declaration of Helsinki. The study was also approved by the medical/ethics review board of Iwai Orthopaedic Medical Hospital. Written informed consent was obtained from all the patients. This study was registered with the UMIN Clinical Trials Registry (UMIN000006957).

### Study interventions

#### Medication

The patients took 900 mg of acetaminophen 3 times a day (total daily amount: 2700 mg). Criteria for discontinuing the medication were ALT/AST ≥ 120 IU/L, total bilirubin ≥ 2.4 mg/dl (E.F. Kuffner), and creatinine clearance ≤ 60.

#### Exercise

**Fig. 1:**
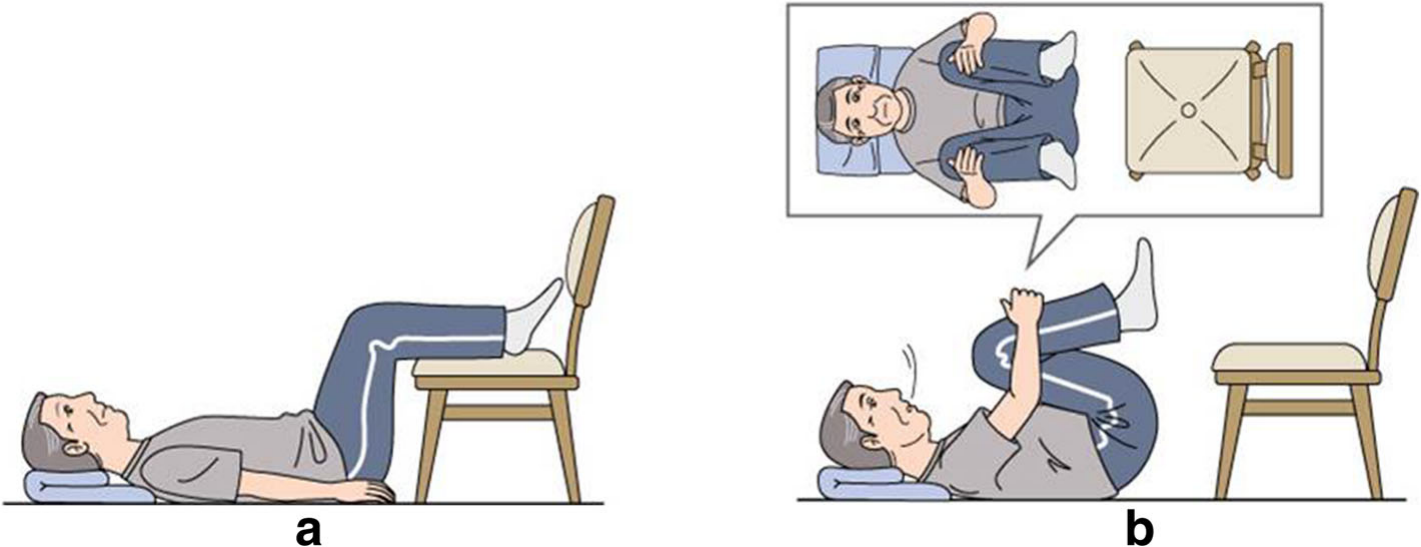
Schema of the exercise. **a**: Lie on your back and put your feet on the chair so that the hip joint and knee are at right angles. **b**: Holding your breath while holding down the knees, slowly count five. On the way, if you get tired, put your feet on the chair and rest

The exercise program included simple back flexion exercises for the first 2 weeks. After receiving instructions from a physical therapist and receiving a manual describing the flexion exercise, the participants performed a total of 6 sets of 10 repeats per day (after getting up, around 10 am, after lunch, around 3 pm, after dinner, and before bedtime). The manual also included evidence-based information on treatment and prevention of LSS, including self-management and risk factors. We illustrated the exercise in Fig. 1.

#### Acupuncture

Therapy was conducted by an acupuncturist who had received postgraduate training in acupuncture and had more than 10 years of clinical experience. It was performed 5 times a month (twice in the first week and once each week from 2 to 4) with acupuncture needles 0.18 mm in diameter and 40 mm in length (Seirin, Japan).

The acupuncture sites were 1) BL-23 (Shenshu): bilateral area 1.5 cm outward from the spinous process of L2, with an insertion depth of 2 cm, 2) BL-25 (Dachangshu): bilateral area 1.5 cm outward from the spinous process of L4, with an insertion depth of 2 cm, 3) BL-5 3(Hoko): bilateral area 9 cm outward from the spinous process of S2, with an insertion depth of 4 cm, 4) BL-54 (Zhibian): bilateral area 9 cm outward from the spinous process of S4, with an insertion depth of 4 cm, 5) BL-40 (Weizhong): middle of the popliteal fossa, 6) GB-34 (Yanglingquan): inferior recess in the fibular head, and 7) BL-57 (Shengshan): lower end of the groove of the inner and outer head of the gastrocnemius [13].

In all three groups, loxoprofen [up to 3 times a day] or celecoxib [up to twice a day] can be used as needed when pain is present. The drug must not be taken on the day of visit for efficacy evaluation and the day before that.

### Outcomes

The primary outcomes were the results of the Zurich claudication questionnaire (ZCQ) before and after 4 weeks of treatment. The ZCQ is a self-administered questionnaire consisting of three different subscales. The first subscale grades symptom severity (1 to 5), the second grades physical function (1 to 4), and the last grades patient’s satisfaction after the treatment (1 to 4). The score increases with worsening disability [14]. We used the linguistically validated Japanese version of the ZCQ, which was developed in our previous study [15].

At study end, an investigator (HO) blinded to individual patient treatment regimens assessed the outcomes.

### Statistical analysis

Statistical analyses were conducted using SAS 9.4 (SAS Institute, Inc., Cary, NC, USA). In previous report, minimally clinically important difference was ≥ 0.5 for both ZCQ Physical Function and ZCQ Symptom Severity [16]. The follow-up assessment was done for 1 month from the first visit. We applied a strict set of criteria (≥ 0.8 points) for the purpose of adjustment of multiple comparisons of 3 groups. The sample size has been calculated to detect a difference between groups of 0.8 points in changes of symptom severity on ZCQ for the pairwise comparisons of each of the three treatments, using Bonferroni correction. This assumption corresponds to our preliminary study. We calculated that with a sample size of 42 patients per group, a total of 126 patients would provide 80% power to detect this difference with P set at 0.0167 (= 0.05/ 3).

Descriptive statistics, including means, standard deviations, and percentages, were used to characterize the study population. Differences in ZCQ scores before and after 4 weeks of treatment were tested using the paired t-test. Least square mean analysis (SAS procedure PROC GLM) was used to assess the following dependent variables in the treatment groups: changes in symptom severity and physical function scores of the ZCQ and patient’s satisfaction after treatment assessed with the ZCQ. Least square means were adjusted for age, sex, body mass index (BMI), smoking status, and baseline ZCQ scores. All statistical tests were 2-tailed. The level of statistical significance was set at *p* < 0.05.

## Results

**Fig. 2:**
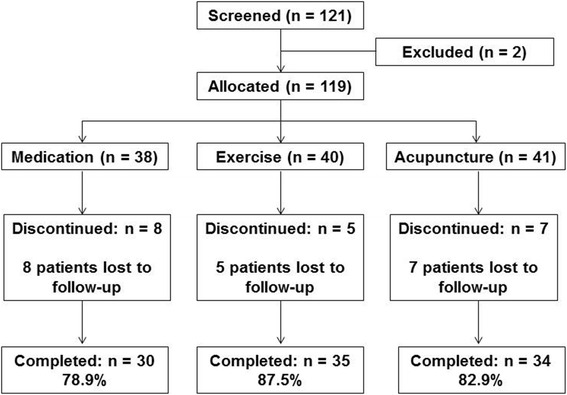
Flowchart of participants in LAP study

One hundred and twenty-one patients were enrolled. Two patients were excluded for positive straight leg raise test. Thirty-eight, 40, and 41 patients were allocated to the medication, exercise, and acupuncture groups, respectively (Fig. 2). The baseline characteristics of these patients are shown in Table 1. There were significant differences in sex, BMI, and smoking status among the groups. Patients who dropped out (medication = 8, exercises = 5, acupuncture = 7) had not been visited since the first visit. These patients can only collect data at the first visit. In the questionnaire at the time of the final follow up, each group was observed to comply with the assigned treatment, and no serious adverse events occurred. Regarding the co-intervention of loxoprofen and celecoxib with regard to pain exacerbation, more than half of the data are missing and cannot be analyzed.

**Table 1:** Baseline characteristics of patient according to the treatment approaches

	Medication (*n* = 38)	Exercise (*n* = 40)	Acupuncture (*n* = 41)	*p*-value
Age	68.7	69.8	70.2	0.64
Sex-Male (%)	24 (63.2)	13 (32.5)	16 (39.0)	0.02
BMI	24.6	23.8	22.4	0.01
Smoking (%)	22 (57.9)	9 (22.5)	14 (34.1)	0.01
Alcohol consumption (%)	13 (34.2)	5 (12.5)	7 (17.1)	0.06
ZCQ symptom severity	3.1	2.8	3.0	0.23
ZCQ physical function	2.3	2.0	2.3	0.06

*BMI* Body Mass Index, *ZCQ* Zurich claudication questionnaire

The mean reduction in ZCQ scores (symptom severity and physical function) and mean ZCQ scores (treatment satisfaction) are reported in Table 2, and between-group differences are reported in Table 3.

**Table 2:** Adjusted mean ZCQ score reductions (symptom severity, physical function) and mean ZCQ scores (surgery satisfaction)

	Medication *n* = 30 mean (95% CI)	Exercise *n* = 35 mean (95% CI)	Acupuncture *n* = 34 mean (95% CI)
Symptom severity	− 0.19 (− 0.47–0.09)	− 0.17 (− 0.44–0.09)	− 0.42 (− 0.69 – − 0.16)
Physical function	− 0.15 (− 0.35–0.06)	0.07 (− 0.12–0.27)	− 2.1 (− 0.40 – − 0.01)
Surgery satisfaction	2.78 (2.50–3.06)	2.48 (2.20–2.76)	2.16 (1.89–2.42)

*ZCQ* Zurich claudication questionnaire, *CI* Confidence Interval

**Table 3:** Between-group differences in ZCQ least-square means adjusted for age, sex, BMI, and confounding factors

	Medication – Exercise		Medication – Acupuncture		Exercise – Acupuncture	
	LS means	*p* - value	LS means	*p* – value	LS means	*p* -v alue
Symptomseverity	− 0.02	0.92	0.23	0.18	0.25	0.14
Physicalfunction	− 0.22	0.09	1.95	0.06	2.17	0.02
Surgerysatisfaction	0.3	0.09	− 0.62	0.0004	0.32	0.06

*ZCQ* Zurich claudication questionnaire

Symptom severity ZCQ scores were improved significantly after treatment in the medication (*p* = 0.048), exercise (*p* = 0.003), and acupuncture (p = 0.04) groups. Physical function ZCQ scores were improved significantly after treatment in the acupuncture (*p* = 0.045) group but not in the medication (*p* = 0.20) and exercise (*p* = 0.29) groups.

The number of patients who decreased ≥ 0.8 points in symptom severity ZCQ scores was 11 (36.7%), 9 (25.7%), 12 (35.3%) for medication, exercise and acupuncture group, respectively. There was no significant difference between the groups (chi-square test *p* = 0.51). The number of patients who decreased ≥ 0.8 points in physical function ZCQ scores was 7 (23.3%), 4 (11.4%), 10 (29.4%) for medication, exercise and acupuncture group, respectively. There was no significant difference between the groups (chi-square test *p* = 0.33).

The mean reduction of the ZCQ physical function score was significantly greater after acupuncture than after exercise. The mean ZCQ score (treatment satisfaction) was significantly greater after acupuncture than after medication.

## Discussion

Acupuncture stimulation led to improvement both in terms of symptom severity and physical function, as well as to high treatment satisfaction, as determined with the ZCQ in patients with L5 radiculopathy. In contrast, medication and flexion exercise improved only symptom severity ZCQ scores. Furthermore, we found that acupuncture was significantly more efficient than physical exercise on the physical function subscale of the ZCQ and than medication on the treatment satisfaction subscale.

Many LSS patients are elderly, and careful consideration must be given to the risk related to the use of analgesic drugs. According to the guideline of the American Geriatrics Society, NSAIDs may lead to gastrointestinal ulcer, renal disorders, hypertension, cardiovascular events, and susceptibility to bleeding due to cyclooxygenase inhibition. The guideline recommends acetaminophen as the first-line drug for low back pain and osteoarthritis of the elderly [17].

There is no RCT on which acetaminophen is used for LSS. However, there are papers describing the possibility that it may be effective for neuropathic pain. Compared with NSAIDs, acetaminophen is superior in safety. Since this drug acts centrally, it is suggested that it may also be effective for neuropathic pain [18].

There were no obvious adverse events including liver dysfunction in this study, suggesting that acetaminophen is safe to use. The dosage of acetaminophen should be carefully adjusted in patients with liver dysfunction or chronic alcohol ingestion. There were no such patients in this study, and therefore a uniform dose was used. Although the analgesic mechanism of acetaminophen has not been fully elucidated, it is known that the inhibitory effect on cyclooxygenases 1 and 2 is very weak [17]. This excludes the inhibition of peripheral prostaglandin synthesis responsible for the anti-inflammatory effect of NSAIDs. Instead, one pain relief mechanism could be via the activation of serotonin in the intrinsic descending pain suppression system. In this regard, the effect of acetaminophen, which shows excellent analgesic action not only in nociceptive but also neuropathic pain, on several central neurotransmitters has been experimentally demonstrated [8]. A recent placebo-controlled trial has shown that acetaminophen is effective not only for physical pain but also for psychogenic pain or anxiety [19, 20]. Epidemiological studies have revealed that many LSS patients complain of psychological distress [21]. Therefore, acetaminophen may alleviate not only nociceptive and neuropathic pain during LSS but also positively affect the psychogenic factors [22].

Although exercise has been combined with medication in patients with LSS, there has been no evidence of its effectiveness for LSS with neurogenic claudication [23]. Typically, flexion relives the symptoms of LSS. Thus, we utilized flexion exercise alone for LSS treatment. Although the efficiency of this approach has not been tested, combinations with other types of physical therapy and multiple exercise programs have been suggested to alleviate lower leg pain and increase the walking distance. It was suggested that flexion exercise mitigate LSS symptoms even after the standard treatment. Therefore, whether flexion exercise is useful at the early onset of LSS deserves further research [24, 25].

In this trial, acupuncture was the most efficient conservative treatment for L5 radiculopathy. It is known that mechanisms of pain suppression are different between acupuncture and local analgesic drugs, such as acetaminophen. The physical stimulus of acupuncture is transmitted to the center via the neural afferent path. As a result, the mechanism of pain suppression is activated. Local analgesic drugs act on sensory nerves and blocks pain transmission. Given the differences in these mechanisms, their efficiencies may also be different. Muscle pain excites nerve fibers, such as C fibers, followed by excitation of the motor nervous system and excessive strain of the muscle, which exacerbates the pain [26]. There are reports that acupuncture stimulation of muscles increases pain threshold and muscle blood flow, which may suppress this excessive strain [27]. Improvement of clinical symptoms and blood flow improvement are related. It was reported that acupuncture affected the blood flow improvement, and one possible mechanism was associated with regulation of systemic vascular resistance via modulation of sympathetic tone [28].

In this study, acupuncture led to higher scores on the treatment satisfaction subscale of the ZCQ. Continuous palpation of the patient’s body by the acupuncturist involves techniques such as touching, stroking, and pushing the painful areas, which may lead to emotional relief [27].

This study has several limitations. First, this was an open-label study in which the assignment was not random but according to the visit time. Furthermore the data of the duration of symptoms has not been collected in this study. Second, only short-term results (4 weeks from intervention) were examined. Further mid- or long-term studies are required to determine the suitability of conservative management of LSS.

## Conclusion

The present study in patients with L5 radiculopathy compared the efficiency of three conservative treatments (medication [acetaminophen], exercise, and acupuncture). Acupuncture was significantly more effective than physical exercise according to the physical function score of the ZCQ and than medication according to the satisfaction score. The present study provides new important information that will aid decision making in LSS treatment.

## Abbreviations

ABI: Ankle brachial pressure index
BMI: Body mass index
CT: Computed tomography
LSS: Lumbar spinal stenosis
MMT: Manual muscle testing
MRI: Magnetic resonance imaging
NSAID: Nonsteroidal anti-inflammatory analgesic
PGB: Pregabalin
QOL: Quality of life
ZCQ: Zurich claudication questionnaire
